# The Sustained Psychological Impact of the COVID-19 Pandemic on Health Care Workers One Year after the Outbreak—A Repeated Cross-Sectional Survey in a Tertiary Hospital of North-East Italy

**DOI:** 10.3390/ijerph182413374

**Published:** 2021-12-19

**Authors:** Antonio Lasalvia, Luca Bodini, Francesco Amaddeo, Stefano Porru, Angela Carta, Ranieri Poli, Chiara Bonetto

**Affiliations:** 1 UOC Psichiatria, Azienda Ospedaliera Universitaria Integrata (AOUI) di Verona, Policlinico ‘G.B. Rossi’, P.le Scuro 10, 37134 Verona, Italy; 2 Section of Psychiatry, Department of Neuroscience, Biomedicine and Movement Sciences, University of Verona, 37129 Verona, Italy; luca.bodini@univr.it (L.B.); francesco.amaddeo@univr.it (F.A.); chiara.bonetto@univr.it (C.B.); 3 UOC Psicosomatica e Psicologia Medica, Azienda Ospedaliera Universitaria Integrata (AOUI) di Verona, Policlinico ‘G.B. Rossi’, P.le Scuro 10, 37134 Verona, Italy; 4 Section of Occupational Medicine, Department of Diagnostics and Public Health, University of Verona and Clinical Unit of Occupational Medicine, Azienda Ospedaliera Universitaria Integrata (AOUI) di Verona, Policlinico ‘G.B. Rossi’, P.le Scuro 10, 37134 Verona, Italy; stefano.porru@univr.it (S.P.); angela.carta@univr.it (A.C.); 5 Hospital Health Directorate, Azienda Ospedaliera Universitaria Integrata (AOUI) di Verona, 37122 Verona, Italy; r.poli@sanita.it

**Keywords:** COVID-19, post-traumatic stress, depression, anxiety, burnout, health care workers, occupational health

## Abstract

This study aimed to evaluate the mental health outcomes of health care workers (HCWs) of the Verona academic hospital trust (Italy) one year after the outbreak of COVID-19 and to identify predicted risk factors. A web-based survey was conducted from mid-April to mid-May 2021 on hospital workers one year after the first evaluation performed during the lock-down phase of the COVID-19 pandemic. Post-traumatic stress, general anxiety, depression, and burnout were assessed by using, respectively, the impact of event scale (IES-R), the self-rating anxiety scale (SAS), the patient health questionnaire (PHQ-9) and the Maslach burnout inventory-general survey (MBI-GS). Multivariate logistic regression analysis was performed to identify factors associated with each of the four mental health outcomes one year after the COVID-19 outbreak. A total of 1033 HCWs participated. The percentage of HCWs scoring above the cut-off increased from 2020 to 2021 in all of the outcome domains (anxiety, 50.1% vs. 55.7, *p* < 0.05; depression, 26.6% vs. 40.6%, *p* < 0.001; burnout, 28.6% vs. 40.6%, *p* < 0.001; chi-square test), with the exception of post-traumatic distress. There was also an increase when stratifying by occupation and workplace, with a greater increase for depression and burnout. Multivariate analysis revealed that, one year after the COVID-19 outbreak, nurses were at the greatest risk of anxiety and depression, whereas residents were at the greatest risk of burnout (in terms of low professional efficacy). Working in intensive care units was associated with an increased risk of developing severe emotional exhaustion and a cynical attitude towards work.

## 1. Introduction

Working in large tertiary hospitals during the COVID-19 pandemic has been found to be stressful or definitely traumatic for many health care workers (HCWs) [1]. A number of studies have consistently found that a relevant proportion of HCWs, especially those at the frontline with critically ill COVID-19 patients, have developed clinically significant symptoms of post-traumatic stress, anxiety, depression, and professional burnout [2,3]. Several factors seem to increase the risk of adverse mental health outcomes among HCWs, including working in intensive care units (ICUs), uncertainty of dealing with an unknown illness, the rapid global spread and significant mortality of the disease and lack of personal protective equipment and effective treatment protocols [4]. However, although the COVID-19 outbreak has been acutely stressful for most HCWs, the longer-term impact of the pandemic is still largely unknown. Most studies published so far have investigated the immediate psychological impact of the pandemic on HCWs [5,6,7,8]. Moreover, findings are still conflicting.

The few studies exploring the short-term impact of the pandemic on HCWs provide conflicting results. A study carried out in Singapore amongst medical residents showed that those who were deployed to the higher-risk National Centre for Infectious Diseases to manage patients with COVID-19 had lower perceived stress at the three-month follow-up [9]. Similarly, a study conducted in Belgium amongst ICU nurses showed that they had improved depression, anxiety, and somatization over a two-month period [10]. In contrast, a study conducted on Argentinean HCWs found a deterioration of self-perceived job performance and increased prevalence of depression and anxiety over a four-month period [11]. Moreover, one large prospective study amongst Chinese frontline HCWs found significantly worse psychiatric status (in terms of somatization, obsessive-compulsiveness, interpersonal sensitivity, depression, anxiety, hostility, phobic anxiety, paranoid ideation and psychoticism) and sleep quality a month after the COVID-19 outbreak [12]. Another longitudinal study conducted in China found significantly higher risks for depression, anxiety and post-traumatic stress disorder (PTSD) symptoms during the outbreak period compared with the stable period of the pandemic a month later [13].

To date, very little information is available on the psychological impact of the pandemic on HCWs over a more prolonged period. Moreover, research has so far addressed only some specific hospital units or some given occupational categories. A recent study conducted in Singapore among health care staff of an emergency department found a significant improvement in anxiety in all professionals, but a significant worsening in depression among doctors over a period of one year [14]. A repeated cross-sectional study on intensive care physicians in a COVID-19 hub hospital in Central Italy reported sustained high levels of occupational stress, anxiety, and depression; low satisfaction; and burnout over one year since the pandemic onset [15].

Based on the above literature, more research is needed to fully understand the long-term impact of the pandemic on the full spectrum of hospital workers and on staff working in the various hospital units (including frontline services). Understanding the enduring occupational and psychological effects of working during the COVID-19 pandemic is important since it involves the well-being of many HCWs and, in turn, the effectiveness and safety of the care provided to patients. The objective of the present study was to assess the psychological impact of the COVID-19 pandemic on the full range of occupational profiles of HCWs working in a large academic hospital in north-east Italy one year after the outbreak and to identify personal and job-related factors that might have increased the risk of developing adverse mental health outcomes one year later.

## 2. Materials and Methods

### 2.1. Study Design

The study described here is part of a repeated two-point cross-sectional survey. Specifically, it represents the second evaluation conducted on the HCWs of the Verona University Hospital Trust. They had first been assessed during the lock-down period of the COVID-19 pandemic (mid-April to mid-May 2020). The survey was promoted and conducted by the Section of Psychiatry at the University of Verona and supported by the Health Directorate of the Verona University Hospital Trust. All staff working in the Verona University Hospital during the COVID-19 pandemic were asked to participate. The sample addressed in the study is composed by those who accepted to participate. HCWs participated in the study on a voluntary basis and were asked to sign a written consent form. The findings of the first assessment are presented in full elsewhere [16,17]. One year after the first assessment (mid-April to mid-May 2021), HCWs of the Verona Academic Hospital Trust were invited to re-assess their psychological status. Similarly to the first assessment, the evaluation carried out in 2021 was made by using self-rated scales hosted on a web-based survey platform (*SurveyMonkey*); participants could complete the online questionnaires by using their PCs, smartphones, or other mobile devices. The study description and the invitation to participate, as well as the link to the online questionnaires, was published in the hospital’s newsletter and sent via e-mail to all hospital workers by the Trust Administration. A reminder for completing the questionnaire was sent to all potential participants after one week. The survey was anonymous, and confidentiality of information was guaranteed. The second online survey required about 15–20 min to be completed.

### 2.2. Setting and Participants

The Verona University Hospital is the second largest hospital trust in Italy in terms of the number of beds and the fifth largest in terms of admissions. The hospital staff comprises 5942 personnel (including nearly 1200 residents of the medical specialty schools at the University of Verona). Beginning on 17 March 2020, the Veneto regional government converted part of the hospital into a ‘COVID-19 hospital’. Thus, dedicated pathways for both suspected and confirmed COVID-19 cases were established within the hospital, as well as in other hospital units located in clearly restricted areas specifically devoted to the treatment of patients with COVID-19. All staff members working in the Verona University Hospital were asked to participate in the study.

### 2.3. Assessment Measures

Post-traumatic distress was assessed by using the Impact of Event Scale-Revised (IES-R) [18], a 22-item self-report instrument that measures subjective distress caused by traumatic events on a 5-point scale from 0 (not at all) to 4 (extremely) during the previous seven days. The scale was slightly adapted for this study: participants were first asked whether they might have experienced a stressful/traumatic event at work related to COVID-19. Three response options were available: (1) ‘Yes, definitely’, (2) ‘Yes, maybe’ and (3) ‘No’. Those responding 1 or 2 were then invited to specify (by writing a free response in their own terms) which kind of stressful/traumatic event it was and were asked to rate how much they were distressed or bothered during the past seven days by each item listed in the IES-R. The maximum score is 88 (worst post-traumatic stress state). We used a cut-off score of 24 for detecting symptoms of post-traumatic distress that deserve clinical attention [19]. For the specific purpose of this study, analysis was conducted only on those participants responding ‘Yes, definitely’ to the entry question. Free responses were analyzed using qualitative content analysis, in which consensus codes were reached by two of the authors (A.L. and F.A.) through an iterative process with preliminary and secondary coding. From this analysis, themes emerged into which responses could be categorized. The same procedure has been also applied in the first assessment.

Symptoms of anxiety were assessed by using the self-rating anxiety scale (SAS) [20] that contains 20 items, each rated on a 5-point scale from 1 (a little of the time) to 4 (most of the time). The maximum score of 80 indicates an extremely high anxiety level and the cut-off score for clinically significant anxiety symptoms is 36 [21].

Symptoms of depression were assessed by using the patient health questionnaire (PHQ-9) [22], a self-rated nine-item scale that asks if the subject has experienced symptoms of depression in the previous two weeks. Subjects are asked to rate how often each symptom occurred: 0 (not at all), 1 (several days), 2 (more than half the days), or 3 (nearly every day). The total PHQ-9 score ranges from 0 (absence of depressive symptoms) to 27 (most severe depressive symptoms). We used a cut-off score of 10 to indicate a condition that potentially deserves clinical attention [23].

Burnout was assessed by using the Maslach burnout inventory-general survey (MBI-GS) [24]. It consists of 16 items constituting three subscales that parallel those of the original MBI: emotional exhaustion (EX; 5 items) covers the experience of both emotional and physical fatigue; cynicism (CY; 5 items) reflects indifference, a detached attitude towards work and active disengagement from work; and professional efficacy (EF; 6 items) consists of feelings of competence, successful achievement and accomplishment in one’s work, which diminishes when burnout is developing. All MBI-GS items are scored on a 7-point rating scale ranging from 0 (never) to 6 (always). We used the only Italian cut-off scores available in the literature for the health care sector (>2.20 for EX, >2.00 for CY and <3.66 for EF). These cut-offs were derived from a large sample of mental health professionals working in the Veneto region [25].

Personal sociodemographic information and job characteristics were also collected, including gender, age, having psychological problems developed before the COVID-19 outbreak requiring specialized help, occupation, and place of work (hospital unit). For the purpose of analysis, the various hospital units were grouped according to the degree of clinical engagement with COVID-19 patients, from most engaged to least engaged: ICUs (that during the lock-down phase were entirely dedicated to critically ill COVID-19 patients), sub-intensive COVID-19 wards (i.e. infectious disease, pulmonary medicine and internal medicine wards specifically dedicated to COVID-19), other frontline services dealing with patients with COVID-19 (i.e. radiology and emergency department), non-COVID-19 wards, laboratory diagnostic services (i.e. laboratory medicine, transfusion medicine, immunology, pathology, microbiology) and administration.

### 2.4. Statistical Analysis

Statistical analyses were carried out by using SPSS 22 and Stata 15. Descriptive statistics are reported as frequencies and percentages. Comparisons between categorical variables were performed by using the chi-square test or Fisher’s exact test where appropriate.

The precision of the proportion estimate for each adverse outcome was determined by calculating the margin of error for the two-sided 95% confidence interval (CI). The proportion of participants for each adverse outcome (post-traumatic distress, anxiety, depression, and burnout domains) in relation to personal (gender and age) and job (occupation, work place and experienced traumatic event) characteristics was explored by stratifying for the aforementioned risk factors. Finally, multivariate logistic regression models for the same outcomes gave adjusted odds ratios (ORs) and 95% CIs. Goodness-of-fit measures were estimated for these models. The alpha level was set to 0.05 for all effects.

## 3. Results

### 3.1. Personal and Job Characteristics

A total of 1033 HCWs completed the online survey in April–May 2021. This sample was similar to the one recruited in April–May 2020 with respect to all personal characteristics and occupational profile. The two samples differed only in workplace, with participants of the second assessment being more frequently employed in ICUs (13.6% vs. 9%), sub-intensive COVID-19 units (15.8% vs. 8.3%) and other frontline services dealing with patients with COVID-19 (11.5% vs. 7.6%) and less in non-COVID-19 wards (40.3% vs. 55%) and laboratory diagnostic services (8.9% vs. 11%) (chi-square test, *p* < 0.001).

Table 1 shows personal and job characteristics of Verona hospital workers participating in the 2021 survey.

Table 2 provides information on treatments for mental health problems that participants reported having had received over the previous year.

Overall, 8% (*n* = 86) reported having had received some form of mental health treatment over the previous year. Most of them (68.6%) were still in treatment at the time of evaluation. Among those seeking help for mental health problems, most (68.6%) received treatment by private practitioners rather than by public services (31.4%). Regarding the specific type of intervention, nearly half reported having had received only psychological therapy, 10% psychopharmacological therapy only and 28% a combined therapy. Mental health treatment was considered beneficial by nearly all HCWs reporting to have been treated.

### 3.2. Mental Health Outcomes 1 Year after the Pandemic Onset

The IES-R, the SAS, the PHQ-9, and the MBI-GS were completed, respectively, by 82.5% (*n* = 335), 90.2% (*n* = 932), 88.8% (*n* = 917) and 86.8% (*n* = 897) of eligible participants. When comparing percentages of completers and non-completers with respect to personal information across the four outcome domains, no staff characteristic was significantly associated with the pattern of response/no response, with the exception of the IES-R, which was completed by a higher percentage of females (85.4% vs. 74.2%; Fisher’s exact test, *p* = 0.018).

Overall, one year after the outbreak onset, 34.8% (*n* = 335) of participants reported having had definitely experienced a stressful/traumatic event related to COVID-19 at work. The frequency distribution of specific traumatic events is given in Table 3. In brief, the most frequent traumatic themes reported by hospital workers in the 2021 assessment were related to dealing with death and dying, demanding work conditions and the fear of infection, reported, respectively, by 31%, 24.6%, and 11% of respondents. It should be noted that the ranking (and frequency) of traumatic experiences reported in the 2021 survey was different from that reported in the previous year.

Among those who reported a COVID-19-related traumatic experience, 72.2% (95% CI 67–77%; margin of error 2.5%) showed symptoms of post-traumatic distress at one year. Moreover, in the overall sample, 55.7% (95% CI 52–59%; margin of error 1.6%) reported symptoms of anxiety and 40.6% (95% CI 37–44%; margin of error 1.6%) reported symptoms of depression one year after the onset of the COVID-19 pandemic. Finally, one year after the onset of the pandemic, 50.4% (95% CI 47–54%; margin of error 1.7%) of participants displayed high emotional exhaustion (EX), 46.2% (95% CI 43–49%; margin of error 1.7%) low professional efficacy (EF) and 35.9% (95% CI 33–39%; margin of error 1.6%) cynicism (CY). Moreover, 38.1% (95% CI 43–49%; margin of error 1.6%) scored beyond the cut-off point in all the three MBI-GS scales, thus displaying a burnout condition.

Stratifying by the main personal and professional characteristics, the proportion of participants scoring above the cut-off points in the various outcome domains differed widely.

As shown in Table 4, women, nurses, and staff working in ICUs or sub-intensive COVID-19 units reported higher percentages of anxiety and depression. Specifically, 61.5% of women displayed severe anxiety and 44.3% displayed severe depression. In addition, 69.5% of nurses showed severe anxiety and 49.8% showed severe depression. HCWs working in ICUs reported symptoms of severe anxiety in 65.8% of cases and of severe depression in 58% of cases; similarly, staff working in sub-intensive COVID-19 wards reported higher percentages of severe anxiety and depression, respectively, in 64.8% and 45.3% of cases. Interestingly, anxiety and depression were more frequent among HCWs who had experienced a COVID-19-related traumatic event at work (76.4% and 62.2%, respectively).

Regarding the MBI-GS scales (Table 5), respondents scoring beyond the cut-off score were more frequent among staff working in both ICUs (EX 71.6%; EF 53.4%; CY 57.8%) and sub-intensive COVID-19 wards (EX 61.7%) and, with respect to the occupational profile, among residents (EX 48.3%; 59.1% EF; CY 33.6%) and nurses (EX 59.8%; EF 47.7%; CY 45.2%). HCWs scoring beyond the cut-off score were also more frequent among those who had experienced a COVID-19-related traumatic event at work (73.5% EX; 47.3% EF; 50.5% CY).

### 3.3. Differences in Adverse Mental Health Outcomes between 2020 and 2021

**Figure 1:**
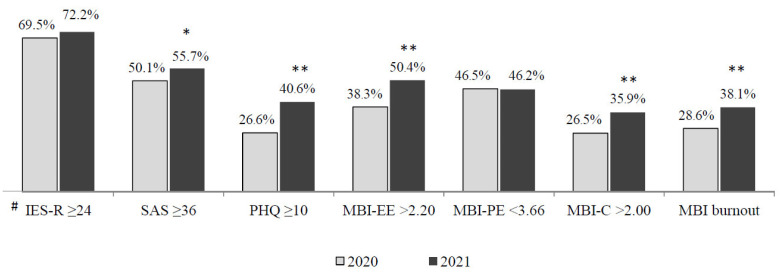
Percentage of respondents scoring above the cut-off score in the various outcome domains across the two cross-sectional assessment points (April–May 2020; April–May 2021) in the overall sample. * *p* < 0.05, ** *p* < 0.001, chi-square test. # Computed only on those who reported having had definitely experienced a traumatic event related to COVID-19 [2020, *n* = 739 (34.3%); 2021, *n* = 335 (34.8%)].

Figure 1 reports the percentages of participants scoring above the cut-off point across the various outcome domains in 2020 and 2021.

**Figure 2:**
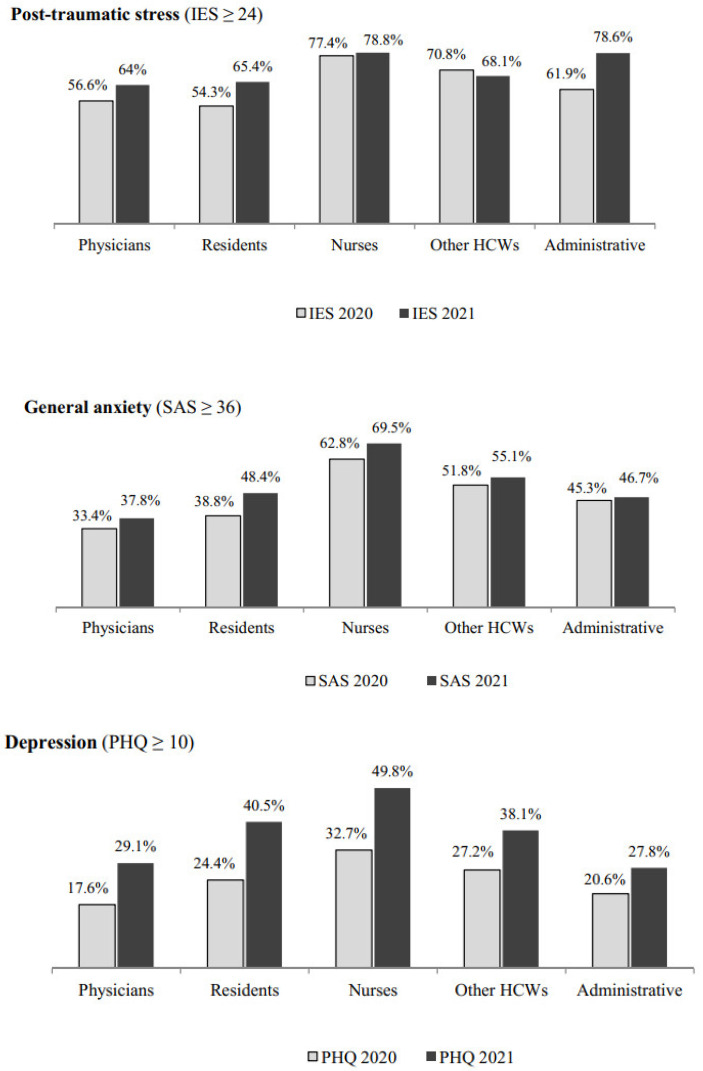

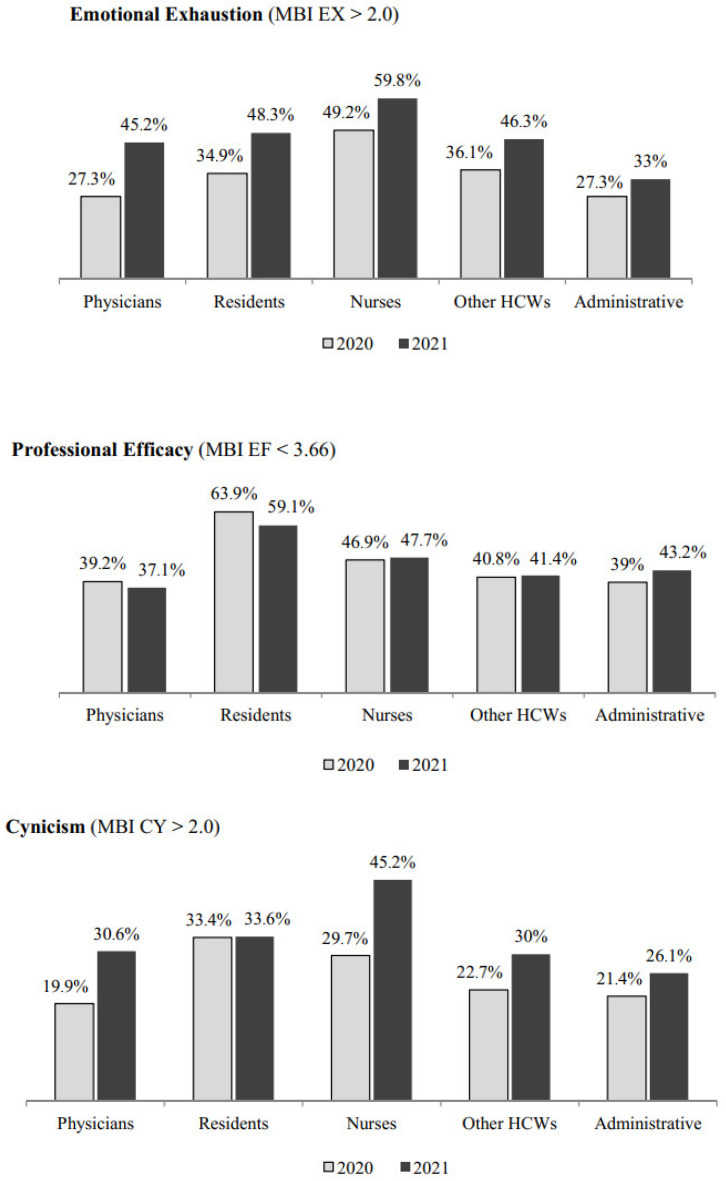
Percentage of respondents scoring above the cut-off score in the various outcome domains across the two cross-sectional assessment points (April–May 2020 and April–May 2021) by occupation.

**Figure 3:**
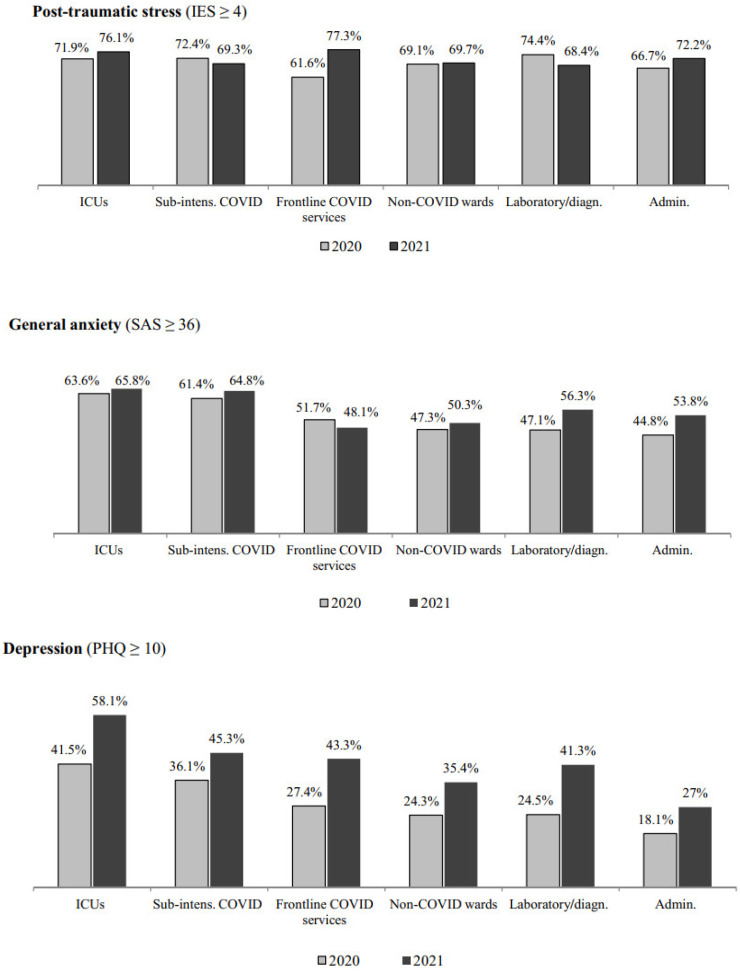

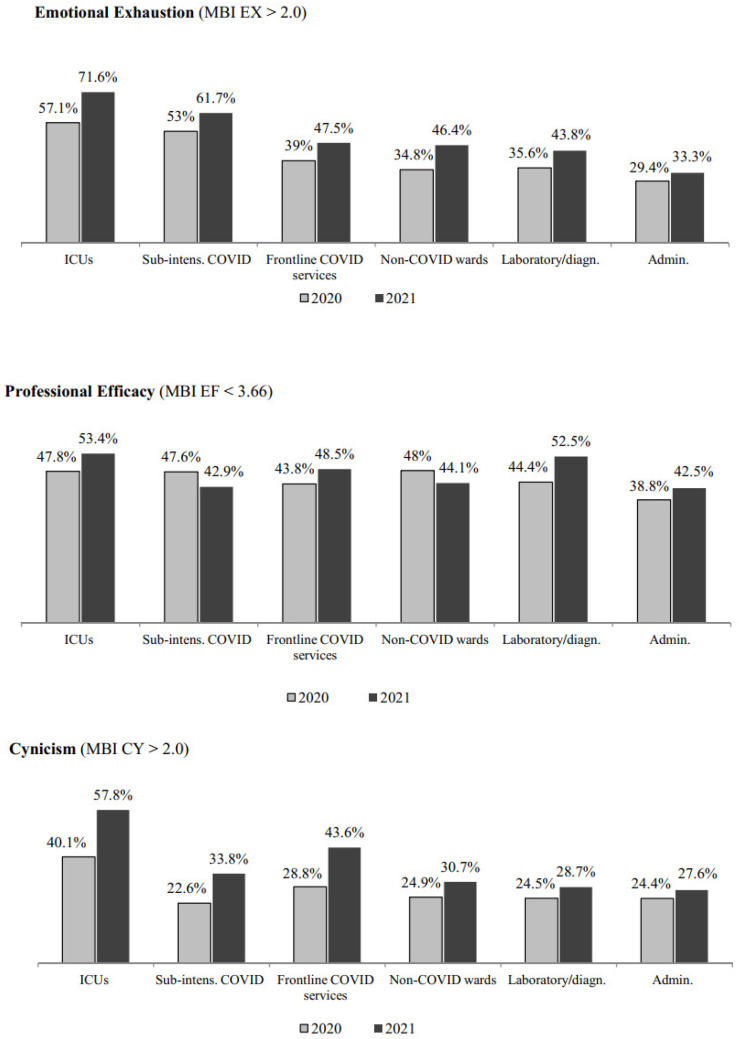
Percentage of respondents scoring above the cut-off score in the various outcome domains across the two cross-sectional assessment points (April–May 2020 and April–May 2021) by workplace.

Overall, the percentage of hospital workers scoring above the cut-off increased from 2020 to 2021 in all the outcome domains considered, except for post-traumatic distress, for which the percentage of those scoring above the cut-off remained substantially similar across the two assessment points (Figure 1). The increase in percentages of participants scoring above the cut-off score across the two assessment points was detected consistently in all the outcome domains when stratified by occupation (Figure 2) and workplace (Figure 3), with a greater increase in the percentages of those showing depression, emotional exhaustion and cynicism.

### 3.4. Risk Factors for Adverse Mental Health Outcomes 1 Year after the Pandemic Onset

Table 6 reports the multivariate regression models for the various psychopathological domains (i.e. post-traumatic distress, anxiety and depression) assessed among Verona hospital workers one year after the onset of the pandemic. Adjusted ORs showed that being older was associated with increased risk of developing severe symptoms of post-traumatic stress (it was the only significant variable). With respect to anxiety, being a woman, a nurse and having had experienced a COVID-19-related traumatic event were associated with an increased risk. Regarding depression, an increased risk was associated with being female, being a nurse or a resident and having had experienced a traumatic event related to COVID-19.

With regard to the MBI-GS (Table 7), working in ICUs (compared with working in the other hospital wards) and having had experienced a COVID-19-related traumatic event increased the risk of developing higher levels of emotional exhaustion and cynicism; being a resident was the only factor that increased the risk of displaying lower professional efficacy.

Overall, being a nurse (compared with being a physician) increased the risk of developing anxiety and depression (adjusted ORs 3 and 2, respectively); being a resident (compared with being a physician) increased the risk of developing anxiety, depression and low professional efficacy (adjusted ORs 1.9, 2, and 2.9, respectively); and having had experienced a COVID-19-related traumatic event increased the risk of anxiety, depression, emotional exhaustion, and cynicism, with adjusted ORs ranging from 2.4 (for cynicism) to 4.4 (for anxiety). Finally, working in ICUs (compared with working in all the other hospital wards) increased the risk of emotional exhaustion and cynicism.

## 4. Discussion

As far as we know, this is the first study that has assessed the psychopathological status of the full range of professional profiles of HCWs working within a large tertiary hospital one year after the onset of the COVID-19 pandemic. Overall, we found that the mental health of HCWs working in a large academic hospital in north-east Italy further deteriorated over the one year since the COVID-19 pandemic onset. The extension of the health care emergency (which required sustained high workload levels within a context of persistent uncertainty on the effectiveness of safety procedures and clinical management protocols) has led to a significant increase in symptoms of depression and burnout among HCWs of the Verona academic hospital. This is consistent with other reports from Italy carried out, for example, on staff working in ICUs [15,26].

The Verona Academic Hospital has been designated a ‘COVID-19 hospital’ since the beginning of the pandemic, and HCWs have continued to provide care to both patients with COVID-19 and all other patients presenting with various medical and surgical conditions in a very difficult context characterized by at least two subsequent pandemic waves (second wave, October 2020–February 2021; third wave, March–May 2021). It should be noted that in Italy, the second and third pandemic waves have been more severe and deadly than the first one [27]. Due to an overall higher number of hospitalized subjects, the second wave of the COVID-19 pandemic had a greater global impact on the health care system in Italy with respect to the first one [28]. In this context, Veneto was one of the northern Italian regions to be more severely affected by the second pandemic wave [29]. On the other hand, after the first pandemic wave, significant changes occurred over the subsequent months within the Italian health care system that improved the response to the pandemic and the confidence of HCWs in dealing with patients with COVID-19. For example, the total number of ICU beds was increased in several Italian hospitals and primary care physicians were involved directly in the initial management of patients COVID-19. Furthermore, there were more nasopharyngeal swabs available to test all subjects with symptoms potentially related to COVID-19 infection [30]. Moreover, from the beginning of January 2021, Italian HCWs were the first occupational category to be offered anti-SARS-CoV-2 vaccination, and from April 2021, the vaccination was made mandatory for all HCWs in order to contain the third wave of the disease.

Interestingly, changes in epidemiological patterns of infection and improvements in the health care system occurred during the first pandemic year are reflected by the ranking of the stressful or traumatic events reported by HCWs participating in this study. One year after the onset of the pandemic, the most frequently reported topic by Verona hospital workers was that of ‘death and dying’ (e.g., having to deal with a great number of deaths in a relatively short time, to see patients dying alone as relatives were not allowed to enter the restricted areas or to communicate by telephone the death of a beloved one to relatives), whereas during the 2020 evaluation it was ranked third (this is consistent with the fact that in Italy the mortality of the COVID-19 pandemic was higher in the second *wave* than in the first one). Similarly to the 2020 evaluation, ‘working under pressure’ remained the second most frequent topic in the 2021 evaluation (burdensome working conditions of HCWs was indeed a major issue throughout the first pandemic year), whereas ‘fear of contagion’ (e.g., fear of being infected with COVID-19 and/or to infect family/relatives or other colleagues at work) was ranked third after one year (probably due to better tracing protocols and improved personal protective measures, including vaccination). However, both advances in diagnostic procedures/clinical management and improvement in protective/preventive measures do not seem to have produced significant effects in mitigating the strain placed by the pandemic on the health care system and reducing adverse mental health outcomes among HCWs. We found that one year after the onset of the pandemic, HCWs (especially nurses, residents, and staff working in ICUs) still reported high levels of post-traumatic distress, anxiety, depression, and burnout. Indeed, our findings indicate that one year after the beginning of the COVID-19 health care emergency the prevalence of depression and burnout (specifically in its components of emotional exhaustion and cynicism) further increased among Verona hospital workers.

Multivariate analyses revealed that nursing staff, irrespective of the hospital unit in which they worked, represent the professional category at greatest risk of developing anxiety and depression. This finding parallels the results obtained from the same hospital population during the first pandemic wave [16]. Nurses are particularly vulnerable to psychological distress in the workplace, as during the COVID-19 pandemic most of them have experienced sudden and dramatic challenges in terms of increased workload, reassignment/redeployment to other roles or duties, infection threat, COVID-19-related traumatic events and frustration with the death of patients for whom they provided care [31]. We also found that sustained engagement with severely (or critically) ill patients with COVID-19 requiring intensive care is a risk factor for developing burnout, specifically in terms of increased emotional exhaustion, and of a detached cynical attitudes towards work and the patients who receive care [32]. A substantial overlapping finding has been reported in the survey conducted at the time of first pandemic wave [17]. Finally, we found that one year after the onset of the pandemic, resident physicians were more severely burdened by burnout than any other professional category. Specifically, they were at higher risk of experiencing a reduced sense of professional efficacy. This is not an unexpected finding: researchers have shown that residents are at increased risk of burnout, in light of the position they have within the organization and the tasks they are generally assigned. This might have been amplified during the COVID-19 pandemic.

It is interesting that only a small proportion of participants in this study (8%) reported having had received some kind of mental health treatment over the previous year. It is worth noting that this proportion is substantially similar to the percentage of respondents who at the time of first assessment reported having had received treatment for mental health problems developed before the outbreak (6%) [16]. This is a relevant finding as the proportion of respondents scoring above the cut-off (for example, in the anxiety (56%) and depressive (41%) domains (reflecting people with a mental health condition that deserves timely and careful clinical attention)) was nearly seven times higher than those reporting having had received mental health treatment over the previous year. This suggests that most HCWs in need of mental health care either did not ask for specialized help or were unwilling to admit having such problems. Reluctance to seek mental health treatment or disclose their own mental health problems is common among health care professionals, particularly physicians [33]. Concerns about stigma contribute to reluctance to seek treatment, which has the effect of delaying treatment, especially among those with the most severe symptoms [34]. Moreover, researchers have reported that HCWs with higher burnout levels are more likely to perceive stigma associated with seeking help for their distress, making them a particularly vulnerable population [35,36]. Strategies to reduce stigma and promote a culture of well-being among health care professionals are needed.

This study has several limitations. The first is the relatively low response rate. However, web-based surveys generally have lower response rates than face-to-face or telephone interviews or mail surveys, and the response rates that have been reported are very similar to our response rate [37]. In addition, some surveys involving physicians had even lower response rates than ours [38]. A second limitation is the sample addressed in this study may be biased since those workers more engaged with critically ill patients with COVID-19 and/or more psychologically distressed have been more likely to participate (thus resulting in an overestimation of HCWs with adverse mental health outcomes). A third limitation is related to the study design. Although the group evaluated in the present study might have been composed of essentially the same subjects assessed during the first pandemic wave (as they were drawn from the same hospital population), the two-time point, cross-sectional design implies that we are unable to follow-up change in individual participants from the first assessment to the second. However, this was unavoidable since we needed to ensure absolute anonymity in the face of stigma and potential compensation lawsuits. A fourth limitation regards the multivariate logistic regression assumptions; the sample size for each regression model is about 900, with the only exception for IES-R (*n* = 335). A general guideline is that a minimum of 10 cases with the least frequent outcome for each independent variable is needed. The IES-R does not respect this rule in one category for occupation (i.e., ‘administrative staff’) and two categories for workplace (i.e., ‘laboratory diagnostic services’; ‘administration’); regarding multi-collinearity among the independent variables, the Chi-square tests between each couple of them showed that they were all significantly correlated (*p* < 0.05), with the exception of (gender, age), (gender, traumatic event) and (age, traumatic event). Finally, the possibility of applying our findings to other nations should be taken with caution, as the study was conducted within the specificity of the Italian national health care system.

Overall, the findings of this study (together with the results obtained from the same hospital population during the first pandemic wave [16,17]) seem to suggest that the psychological reaction of HCWs to the challenge posed by the COVID-19 outbreak may be different according to the specific stage of the pandemic. At the beginning (during the first wave), an ‘acute stress’ reaction was observed, characterized by, for example, post-traumatic response, fear of contagion, and anxiety. One year after the pandemic onset (after having had faced enduring and challenging work conditions determined by the second and third pandemic waves), a ‘chronic stress’ reaction seems to have emerged, characterized by depressive and burnout responses. This ‘chronic’ job-related stress reaction displayed by HCWs represents a relevant issue, both in terms of personal suffering and of reduced quality and safety of care provided to patients.

Health care systems will need to address the psychological impact of the pandemic on HCWs by monitoring reactions and performance, paying careful attention to assignments and schedules, modifying expectations, assessing occupational risk, promoting effective work-life balance support mechanisms, and providing psychological support services for those more in need of mental health care [39]. However, specific types of intervention will depend on the stage of the pandemic and the specific mental health care needs displayed by HCWs.

As a future development of this research, we are designing an intervention study on hospital staff at greater risk of adverse psychological outcomes (e.g., nurses, residents, and those working in ICUs). The implementation will comprise mindfulness-based stress reduction protocols that have had some promising effects on burnout of frontline HCWs during the current COVID-19 pandemic [40].

## 5. Conclusions

The mental health of health care professionals has further deteriorated in the year since the COVID-19 pandemic onset. The long duration of the pandemic has exposed HCWs to an unprecedented strain. Excessive and prolonged workload, isolation, uncertainty about clinical management protocols, and safety measures have resulted in sustained psychological distress among hospital workers that should be expeditiously addressed by health care administrations.

## Figures and Tables

**Table 1:** Personal and job characteristics of participants assessed in April–May 2021 (*n* = 1033).

	*n*	%
**Gender** (19 missing)		
Male	234	23.1
Female	780	76.9
**Age** (years) (3 missing)		
<36	332	32.2
36–55	503	48.8
>55	195	18.9
**Occupation** (11 missing)		
Physicians	138	13.5
Residents	171	16.7
Nurses	379	36.7
Other health care staff	233	22.6
Administrative staff	101	9.8
**Work place** (40 missing)		
Intensive care units	135	13.6
Sub-intensive COVID-19 wards ^1^	157	15.8
Frontline services dealing with COVID-19 ^2^	114	11.5
Non-COVID-19 wards	400	40.3
Laboratory diagnostic services ^3^	88	8.9
Administration	99	10.0

^1^ Infectious Disease Unit, Pulmonary Medicine, Internal Medicine units converted to COVID-19 units; ^2^ Radiology and Emergency Departments; ^3^ Laboratory Medicine, Transfusion Medicine, Immunology, Pathology, Microbiology.

**Table 2:** Treatment for mental health (MH) problems reported by participants assessed in April–May 2021 (*n* = 1033).

.	*n*	%
**HCWs having had received MH treatment** (10 missing)		
Yes	86	8.4
No	937	91.6
**HCWs still in MH treatment** (10 missing)		
Yes	59	68.6
No	27	31.4
**Type of mental health provider** (10 missing)		
Private	59	68.6
Public	27	31.4
**Type of MH intervention received** (10 missing)		
Pharmacological treatment	9	10.5
Psychotherapy	40	46.5
Both	24	27.9
Other	13	15.1
**Perceived efficacy of MH treatment** (11 missing)		
Yes	76	89.4
No	9	10.6

HCWs, health care workers.

**Table 3:** Comparison of traumatic events reported by respondents in the first (April–May 2020) and second (April–May 2021) assessment points.

	First Assessment			Second Assessment			
	*n*	%	Rank	*n*	%	Rank	Δ Rank
High number of deaths, patients dying alone, use of telephone to communicate the death of loved ones	73	18.9	3	71	31.1	1	+2
Feeling under pressure due to time and staff constraints	102	26.4	1	56	24.6	2	−1
Fear of being infected and/or infecting others	82	21.2	2	25	11.0	3	−1
Insufficient supervision, unclear guidelines, shortage of PPE	44	11.4	4	20	8.8	4	0
Reassigned to a COVID-19 unit	31	8.0	5	15	6.6	5	0
Infection or death of a relative, friend or colleague	13	3.4	8	14	6.1	6	+2
Difficult ethical decisions in a short time	16	4.1	6	12	5.3	7	−1
Being infected with COVID-19	14	3.6	7	9	3.9	8	−1
Difficulty of balancing work and family life	11	2.8	9	6	2.6	9	0
	386	100.0		228	100.0		

**Table 4:** Participants scoring below and above the cut-off scores in the various psychopathological domains (IES-R, SAS and PHQ) at the second assessment point (April-May 2021) by main personal and professional characteristics.

	Post-Traumatic Distress * (*n* = 335)			Anxiety (*n* = 932)			Depression (*n* = 917)		
	<24 IES-R *n* (%)	≥24 IES-R *n* (%)	*p*	<36 SAS *n* (%)	≥36 SAS *n* (%)	*p*	<10 PHQ *n* (%)	≥10 PHQ *n* (%)	*p*
**Gender**									
Male	22 (31.9)	47 (68.1)	0.364	129 (62.9)	76 (37.1)	<0.001	142 (70.0)	61 (30.0)	<0.001
Female	69 (26.1)	195 (73.9)		273 (38.5)	437 (61.5)		389 (55.7)	309 (44.3)	
**Age** (years)									
<36	41 (36.9)	70 (63.1)	0.030	146 (47.7)	160 (52.3)	0.038	176 (58.7)	124 (41.3)	0.321
36–55	38 (22.8)	129 (77.2)		177 (39.9)	267 (60.1)		252 (57.8)	184 (42.2)	
>55	14 (24.6)	43 (75.4)		88 (48.9)	92 (51.1)		115 (64.2)	64 (35.8)	
**Occupation**									
Physicians	18 (36.0)	32 (64.0)	0.143	79 (62.2)	48 (37.8)	<0.001	90 (70.9)	37 (29.1)	<0.001
Residents	18 (34.6)	34 (65.4)		80 (51.6)	75 (48.4)		91 (59.5)	62 (40.5)	
Nurses	31 (21.2)	115 (78.8)		102 (30.5)	232 (69.5)		164 (50.2)	163 (49.8)	
Other health care staff	22 (31.9)	47 (68.1)		96 (44.9)	118 (55.1)		130 (61.9)	80 (38.1)	
Administrative staff	3 (21.4)	11 (78.6)		49 (53.3)	43 (46.7)		65 (72.2)	25 (27.8)	
**Work place**									
Intensive care units	16 (23.9)	51 (76.1)	0.870	41 (34.2)	79 (65.8)	0.006	49 (41.9)	68 (58.1)	<0.001
Sub-intensive COVID-19 wards ^1^	27 (30.7)	61 (69.3)		50 (35.2)	92 (64.8)		75 (54.7)	62 (45.3)	
Frontline services dealing with COVID-19 ^2^	10 (22.7)	34 (77.3)		54 (51.9)	50 (48.1)		59 (56.7)	45 (43.3)	
Non-COVID-19 wards	27 (30.3)	62 (69.7)		178 (49.7)	180 (50.3)		228 (64.6)	125 (35.4)	
Laboratory diagnostic services ^3^	6 (31.6)	13 (68.4)		35 (43.8)	45 (56.3)		47 (58.8)	33 (41.3)	
Administration	5 (27.8)	13 (72.2)		42 (46.2)	49 (53.8)		65 (73.0)	24 (27.0)	
**Experienced traumatic event**									
Yes	93 (27.8)	242 (72.2)	-	78 (23.6)	253 (76.4)	<0.001	123 (37.8)	202 (62.2)	<0.001
No	-	-		335 (55.7)	266 (44.3)		422 (71.3)	170 (28.7)	

* Completed only by participants who experienced a COVID-19-related traumatic event; ^1^ Infectious Disease Unit, Pulmonary Medicine, Internal Medicine units converted specifically to COVID-19; ^2^ Radiology and Emergency Departments; ^3^ Laboratory Medicine, Transfusion Medicine, Immunology, Pathology, Microbiology; IES-R, impact of event scale-revised; PHQ, patient health questionnaire; SAS, self-rating anxiety scale.

**Table 5:** Participants scoring below and above the cut-off scores in the various MBI-GS subscales at the second assessment point (April–May 2021) by main personal and professional characteristics.

	Emotional Exhaustion (*n* = 897)			Professional Efficacy (*n* = 897)			Cynicism (*n* = 897)		
	≤2.20 *n* (%)	>2.20 *n* (%)	*p*	≥3.66 *n* (%)	<3.66 *n* (%)	*p*	≤2.00 *n* (%)	>2.00 *n* (%)	*p*
**Gender**									
Male	113 (56.5)	87 (43.5)	0.016	113 (56.5)	87 (43.5)	0.404	126 (63.0)	74 (37.0)	0.791
Female	319 (46.8)	362 (53.2)		362 (53.2)	319 (46.8)		436 (64.0)	245 (36.0)	
**Age** (years)									
<36	146 (49.3)	150 (50.7)	0.158	145 (49.0)	151 (51.0)	0.063	189 (63.9)	107 (36.1)	0.664
36–55	201 (47.4)	223 (52.6)		231 (54.5)	193 (45.5)		267 (63.0)	157 (37.0)	
>55	98 (56.0)	77 (44.0)		105 (60.0)	70 (40.0)		117 (66.9)	58 (33.1)	
**Work place**									
Intensive care units	33 (28.4)	83 (71.6)	<0.001	54 (46.6)	62 (53.4)	0.342	49 (42.2)	67 (57.8)	<0.001
Sub-intensive COVID-19 wards ^1^	51 (38.3)	82 (61.7)		76 (57.1)	57 (42.9)		88 (66.2)	45 (33.8)	
Wards/services dealing with COVID-19 ^2^	53 (52.5)	48 (47.5)		52 (51.5)	49 (48.5)		57 (56.4)	44 (43.6)	
Non-COVID-19 wards	185 (53.6)	160 (46.4)		193 (55.9)	152 (44.1)		239 (69.3)	106 (30.7)	
Laboratory diagnostic services ^3^	45 (56.3)	35 (43.8)		38 (47.5)	42 (52.5)		57 (71.3)	23 (28.7)	
Administration	58 (66.7)	29 (33.3)		50 (57.5)	37 (42.5)		63 (72.4)	24 (27.6)	
**Occupation**									
Physicians	68 (54.8)	56 (45.2)	<0.001	78 (62.9)	46 (37.1)	0.002	86 (69.4)	38 (30.6)	<0.001
Residents	77 (51.7)	72 (48.3)		61 (40.9)	88 (59.1)		99 (66.4)	50 (336)	
Nurses	130 (40.2)	193 (59.8)		169 (52.3)	154 (47.7)		177 (54.8)	146 (45.2)	
Other health care staff	109 (53.7)	94 (46.3)		119 (58.6)	84 (41.4)		142 (70.0)	61 (30.0)	
Administrative staff	59 (67.0)	29 (33.0)		50 (56.8)	38 (43.2)		65 (73.9)	23 (26.1)	
**Experienced traumatic event**									
No	361 (62.2)	219 (37.8)	<0.001	316 (54.5)	264 (45.5)	0.605	418 (72.1)	162 (27.9)	<0.001
Yes	84 (26.5)	233 (73.5)		167 (52.7)	150 (47.3)		157 (49.5)	160 (50.5)	

^1^ Infectious Disease Unit, Pulmonary Medicine, Internal Medicine units converted specifically to COVID-19; ^2^ Radiology and Emergency Departments; ^3^ Laboratory Medicine, Transfusion Medicine, Immunology, Pathology, Microbiology; MBI-GS, Maslach burnout inventory-general survey.

**Table 6:** Multivariate logistic regressions for post-traumatic distress, anxiety, and depression assessed among Verona hospital workers one year after the onset of the pandemic (*n* = 1033).

	Post-Traumatic Distress			Anxiety			Depression		
	Adj OR (95% CI)	*p*		Adj OR (95% CI)	*p*		Adj OR (95% CI)	*p*	
		Category	Overall LR Test		Category	Overall LR Test		Category	Overall LR Test
**Gender**									
Male	1		0.300	1		<0.001	1		0.007
Female	1.40 (0.74–2.66)	0.296		2.40 (1.66–3.47)	<0.001		1.68 (1.15–2.45)	0.007	
**Age** (years)									
<36	1		0.009	1		0.130	1		0.569
36–55	2.86 (1.42–5.76)	0.003		1.45 (0.95–2.22)	0.082		1.25 (0.82–1.91)	0.290	
>55	3.05 (1.25–7.44)	0.014		1.08 (0.65–1.80)	0.762		1.19 (0.71–1.99)	0.510	
**Work place**									
Intensive care units	1		0.441	1		0.094	1		0.099
Sub-intensive COVID-19 wards	0.53 (0.24–1.18)	0.119		0.95 (0.53–1.70)	0.856		0.50 (0.29–0.88)	0.015	
Frontline wards/services	0.89 (0.34–2.30)	0.805		0.58 (0.31–1.06)	0.074		0.65 (0.36–1.17)	0.149	
Non-COVID-19 wards	0.51 (0.23–1.14)	0.103		0.86 (0.52–1.43)	0.569		0.57 (0.35–0.92)	0.022	
Laboratory diagnostic services	0.47 (0.13–1.70)	0.252		1.25 (0.62–2.54)	0.535		0.78 (0.39–1.56)	0.488	
Administration	0.31 (0.06–1.56)	0.156		1.79 (0.74–4.32)	0.197		0.40 (0.17–0.96)	0.041	
**Occupation**									
Physician	1		0.203	1		<0.001	1		0.034
Resident	2.04 (0.74–5.65)	0.170		1.88 (1.00–3.54)	0.051		2.00 (1.04–3.81)	0.036	
Nurse	2.03 (0.92–4.46)	0.078		3.08 (1.87–5.07)	<0.001		2.14 (1.30–3.54)	0.003	
Other health care staff	1.07 (0.45–2.51)	0.882		1.65 (0.98–2.78)	0.061		1.42 (0.83–2.44)	0.200	
Administrative staff	3.69 (0.54–25.15)	0.182		0.99 (0.42–2.31)	0.974		1.58 (0.67–3.75)	0.296	
**Experienced traumatic event**									
No	-	-	-	1		<0.001	1		<0.001
Yes	-	-		4.42 (3.13–6.25)	<0.001		3.74 (2.72–5.14)	<0.001	
**Number of observations**	319			874			860		
**LR test**	χ^2^(12) = 18.94			χ^2^(13) = 165.58			χ^2^(13) = 120.02		
***p***	0.090			<0.001			<0.001		
**Pearson goodness-of-fit**									
**Number of covariate patterns**	77			170			170		
**χ^2^(df)**	χ^2^(64) = 62.36			χ^2^(156) = 174.11			χ^2^(156) = 169.31		
***p***	0.535			0.153			0.220		
**Area under ROC curve**	0.641			0.742			0.709		

Adj OR, adjusted odds ratio; CI, confidence interval; df, degrees of freedom; LR, linear regression; ROC, receiver operating characteristics.

**Table 7:** Multivariate logistic regressions for the MBI-GS domains (emotional exhaustion, professional efficacy and cynicism) assessed among Verona hospital workers one year after the onset of the pandemic (*n* = 1033).

	Emotional Exhaustion			Professional Efficacy			Cynicism		
	Adj OR (95% CI)	*p*		Adj OR (95% CI)	*p*		Adj OR (95% CI)	*p*	
		Category	Overall LR Test		Category	Overall LR Test		Category	Overall LR Test
**Gender**									
Male	1		0.097	1		0.492	1		0.589
Female	1.36 (0.94–1.96)	0.098		1.13 (0.80–1.59)	0.492		0.90 (0.63–1.30)	0.588	
**Age** (years)									
<36	1		0.364	1		0.564	1		0.619
36–55	1.29 (0.85–1.96)	0.235		1.22 (0.82–1.80)	0.329		1.15 (0.75–1.76)	0.511	
>55	1.04 (0.63–1.72)	0.880		1.07 (0.66–1.73)	0.783		1.29 (0.77–2.16)	0.329	
**Work place**									
Intensive care units	1		0.037	1		0.152	1		0.004
Sub-intensive COVID-19 wards	0.56 (0.31–1.01)	0.053		0.68 (0.40–1.15)	0.155		0.35 (0.20–0.61)	<0.001	
Frontline wards/Services	0.39 (0.21–0.72)	0.003		0.88 (0.58–1.54)	0.660		0.64 (0.37–1.13)	0.123	
Non-COVID-19 wards	0.50 (0.30–0.83)	0.008		0.71 (0.44–1.12)	0.142		0.43 (0.30–0.70)	0.001	
Laboratory diagnostic services	0.44 (0.22–0.88)	0.021		1.32 (0.68–2.54)	0.407		0.48 (0.24–0.96)	0.037	
Administration	0.34 (0.14–0.81)	0.015		0.68 (0.30–1.50)	0.339		0.46 (0.20–1.08)	0.076	
**Occupation**									
Physician	1		0.585	1		0.001	1		0.115
Resident	1.21 (0.65–2.27)	0.542		2.95 (1.62–5.37)	<0.001		1.33 (0.70–2.54)	0.384	
Nurse	1.40 (0.86–2.28)	0.178		1.56 (0.98–2.49)	0.060		1.70 (1.04–2.79)	0.036	
Other health care staff	1.12 (0.66–1.88)	0.676		1.01 (0.61–1.67)	0.971		1.06 (0.62–1.82)	0.835	
Administrative staff	0.90 (0.39–2.08)	0.815		1.52 (0.69–3.33)	0.299		1.02 (0.43–2.43)	0.960	
**Experienced traumatic event**									
No	1		<0.001	1		0.895	1		<0.001
Yes	3.91 (2.82–5.42)	<0.001		1.02 (0.75–1.39)	0.895		2.41 (1.75–3.30)	<0.001	
**Number of observations**	842			842			842		
**LR test**	χ^2^(13) = 127.09			χ^2^(13) = 29.03			χ^2^(13) = 72.51		
***p***	<0.001			0.006			<0.001		
**Pearson goodness-of-fit**									
**Number of covariate patterns**	169			169			169		
**χ^2^(df)**	χ^2^(155) = 166.68			χ^2^(155) = 157.00			χ^2^(155) = 161.08		
***p***	0.247			0.440			0.352		
**Area under ROC curve**	0.717			0.606			0.668		

## Data Availability

Data will be available upon reasonable request. Data will be deposited in the university repository, where reasonable requests for access can be submitted. Data access can be requested from the corresponding author.
